# Factors Affecting De Novo Urinary Retention after Holmium Laser Enucleation of the Prostate

**DOI:** 10.1371/journal.pone.0084938

**Published:** 2014-01-21

**Authors:** Sung Han Kim, Changwon Yoo, Minsoo Choo, Jae-Seung Paick, Seung-June Oh

**Affiliations:** 1 Department of Urology, National Cancer Center, Goyang, Gyeonggi, South Korea; 2 Departments of Biostatistics, Robert Stempel College of Public Health and Social Work, Florida International University, Miami, Florida, United States of America; 3 Department of Urology, Seoul National University Hospital, Seoul, South Korea; Oklahoma University Health Sciences Center, United States of America

## Abstract

**Objective:**

Patients can experience urinary retention (UR) after Holmium laser enucleation of the prostate (HoLEP) that requires bladder distension during the procedure. The aim of this retrospective study is to identify factors affecting the UR after HoLEP.

**Materials and Methods:**

336 patients, which underwent HoLEP for a symptomatic benign prostatic hyperplasia between July 2008 and March 2012, were included in this study. Urethral catheters were routinely removed one or two days after surgery. UR was defined as the need for an indwelling catheter placement following a failure to void after catheter removal. Demographic and clinical parameters were compared between the UR (n = 37) and the non-urinary retention (non-UR; n = 299) groups.

**Results:**

The mean age of patients was 68.3 (±6.5) years and the mean operative time was 75.3 (±37.4) min. Thirty seven patients (11.0%) experienced a postoperative UR. UR patients voided catheter free an average of 1.9 (±1.7) days after UR. With regard to the causes of UR, 24 (7.1%) and 13 (3.9%) patients experienced a blood clot-related UR and a non-clot related UR respectively. Using multivariate analysis (p<0.05), we found significant differences between the UR and the non-UR groups with regard to a morcellation efficiency (OR 0.701, 95% CI 0.498–0.988) and a bleeding-related complication, such as, a reoperation for bleeding (OR 0.039, 95% CI 0.004–0.383) or a transfusion (OR 0.144, 95% CI 0.027–0.877). Age, history of diabetes, prostate volume, pre-operative post-void residual, bladder contractility index, learning curve, and operative time were not significantly associated with the UR (p>0.05).

**Conclusions:**

De novo UR after HoLEP was found to be self-limited and it was not related to learning curve, patient age, diabetes, or operative time. Efficient morcellation and careful control of bleeding, which reduces clot formation, decrease the risk of UR after HoLEP.

## Introduction

Holmium laser enucleation of the prostate (HoLEP) is a newer surgical treatment of benign prostatic hyperplasia (BPH) that was introduced in 1995. It involves enucleation and morcellation procedures [1]. HoLEP enables any size of prostate to be treated in a minimally invasive manner [2]–[4]. Many authors have reported that HoLEP is as effective as the transurethral resection of prostate with much shorter duration of urethral catheterization [5]–[9]. However, sometimes after urethral catheter removal, clinicians encounter urinary retention (UR) resulting in the need for a re-catheterization due to a voiding failure.

To avoid a bladder injury during morcellation, it is required to keep the bladder distended. Therefore, due to over-distention of the bladder, there is a concern for myogenic injury of the bladder that is responsible for de novo UR, despite a successful relief of a bladder outlet obstruction. However, no report has been previously published on de novo UR after HoLEP, however, a few reports have mentioned de novo UR is a postoperative complication of prostatectomy [10], [11]. This study was undertaken to describe the characteristics of de novo UR, and to identify independent risk factors that influence UR.

## Materials and Methods

### Ethics statement

This retrospective study was approved by the Institutional Review Board of the Seoul National University Hospital (IRB approval No. H1301-049-461). Written informed consents from the patients were not required.

### Patient population

The study cohort comprised 336 patients that underwent HoLEP for symptomatic BPH by two surgeons (SJO, JSP) between July 2008 and March 2012. All medical records in our prospectively collected database were reviewed. The inclusion criteria were lower urinary tract symptoms (LUTS) that suggest patients have BPH and an age over 50 years. The exclusion criteria were a baseline history of UR, prostate surgery, urethral stricture, genitourinary malignancy, neurogenic bladder, urinary tract infection, or a congenital genitourinary anomaly.

All patients underwent a baseline evaluation including: history taking, physical examination, International Prostate Symptom Score (IPSS), uroflowmetry (UFM), postvoid residual urine volume (PVR) measurement, urinalysis, serum creatinine, serum prostate-specific antigen (PSA), and transrectal ultrasonography (TRUS). A multichannel urodynamic study (MMS UD-2000, Medical Measurement System, Ennschede, Netherlands) was performed to help differentiate a bladder outlet obstruction and a detrusor overactivity. If necessary, a TRUS-guided prostate biopsy was carried out for those suspected prostate cancer.

### Surgical procedure and follow-up

The surgical indications for HoLEP included moderate to severe LUTS refractory to medication. The HoLEP procedures used were as previously described in our papers [12], [13]. The following intraoperative variables were documented; total operative time (including enucleation and morcellation), total energy and power used, intraoperative complications, and enucleated prostatic weight. At the end of surgery, a 22 Fr three-way urethral Foley catheter was placed, and its balloon was inflated with 30 ml of saline. Retrieved tissues were forwarded for histopathological evaluation. All BPH-related medications were discontinued after HoLEP, and only antibiotics were administered before HoLEP for prophylaxis.

Urethral catheters were typically removed at postoperative one or two days after confirming clear urine color without significant gross hematuria. Patients were instructed to void within three to four hours after catheter removal. Particular attention was paid to check PVR to make sure successful voiding accomplished. Patients were discharged when the PVRs of two consecutive voiding were less than 100 ml. Those with PVR of 100 ml or more were encouraged to void repeatedly every three to four hours. If patients failed to void, indwelling urethral catheter was placed. They were instructed to visit the outpatient clinic for a voiding trial, usually five to seven days later for delayed trial of emptying after catheter removal. UR was defined as the need for an indwelling catheter placement following a failure to void after initial voiding trial at one or two days after operation. After HoLEP, the subjective and objective treatment outcomes were followed at 2 weeks, 3, 6 and 12 month postoperatively with IPSS, UFM, and PVR.

### Statistical analysis

Demographic and clinical parameters in UR and non-UR groups, including intra-operative and peri-operative periods, were compared. Morcellation efficiency was defined as enucleated prostate weight divided by morcellation time [14]. Continuous variables were analyzed using the t-test and the Mann-Whitney test, and nominal and categorical variables using the Chi-square test and Fisher's exact test. Only those variables found to be clinically and statistically significant by univariate analysis were included into the multivariate analysis conducted to identify risk factors for de novo postoperative UR. A 5% level of significance was used. A statistical analysis was performed using SPSS for Windows ver. 18.0 (SPSS Inc., Chicago, IL).

## Results

Data were obtained from the medical records of 336 patients who underwent HoLEP. The mean overall patient age was 68.3 (±6.5) years and the mean LUTS duration was 27.1 (±6.0) months. Other preoperative clinical characteristics including demographics and urodynamics are shown in the Table 1.

**Table 1:** Baseline demographics (n = 336).

Clinical Parameters	Mean ± SD or No. patients (%)
Age (yr)	68.3±6.5
Body mass index (kg/m^2^)	24.1±2.8
LUTS duration (mo)	27.1±6.0
Comorbidities (n, %)	
Hypertension	152 (45.2)
Diabetes	54 (16.1)
Neurological disease	35 (10.4)
Cardiovascular disease	25 (7.4)
PSA (ng/ml)	3.5±4.0
Total prostate volume (ml)	55.6±23.6
Transitional zone volume (ml)	29.7±19.5
Urodynamic parameters	
MUCP (cmH_2_O)	75.8±26.8
Maximal cystometric capacity (ml)	375.1±125.0
Bladder outlet obstruction index	44.0±19.1
PdetQmax (cmH_2_O)	60.6±27.2
Operative parameters	
Total operating time (min)	75.3±37.4
Enucleation time (min)	56.2±25.1
Morcellation time (min)	11.3±9.5
Weight of tissue retrieved (gm)	20.8±17.0
Postoperative parameters	
Duration of catheterization (day)	1.9±1.7
Hospital stay (day)	2.9±1.5

LUTS, lower urinary tract symptoms; MUCP, maximal urethral closing pressure; PdetQmax, detrusor pressure at maximal flow.

Uroflowmetric data and IPSS scores, which included a quality of life (QoL component) showed that HoLEP was very effective in improving LUTS (p<0.001, Table 2). Follow-up IPSS and QoL scores up to 12 months showed gradual improvements in symptoms till 6 months postoperatively and the maintenance of these improvements at 12 months (not shown in the table).

**Table 2:** Changes of outcome parameters in the 336 patients.

Clinical Parameters	Baseline	Post op. 2 week	p-value*
No. of patients	366	283	
IPSS (mean ± SD)			
Obstructive symptom score	10.6±5.8	4.7±4.8	<0.001
Storage symptom score	7.1±3.9	6.2±4.1	<0.001
Total symptom score	17.6±8.8	10.8±7.9	<0.001
Quality of life score	4.1±1.2	2.8±1.7	<0.001
Uroflowmetry and post void residual			
Peak flow rate (ml/sec)	10.3±4.5	18.7±9.8	<0.001
Postvoid residual (ml)	72.2±100.6	22.9±32.8	<0.001

* , paired *t*-test.

Thirty seven patients (11.0%) displayed UR. Among them, 24 patients (7.1%) had clot-related UR in which UR was regarded as outflow obstruction caused by a bloody clot, and 13 patients (3.9%) had non-clot related retention. All patients who had urethral catheter indwelling due to failure to void during hospitalization voided successfully at delayed voiding trial in outpatient visit after discharge. The mean urethral catheter duration was 1.9 (±1.7) days.

No significant differences were found between the UR and the non-UR groups with respect to baseline demographics or perioperative parameters with the exceptions of morcellation efficiency (UR 1.6±0.8 vs. non-UR 2.1±1.3 gm/min, p<0.001), reoperation due to bleeding (UR 4/37 vs. non-UR 1/299, p = 0.001), and transfusion (UR 3/37 vs. non-UR 3/299, p = 0.02) (Table 3). The multivariate analysis of variables found significant by univariate analysis also showed that the morcellation efficiency, reoperation due to bleeding, and transfusion were significantly independent factors of UR (Table 3).

**Table 3:** Comparison between non- urinary retention (non-UR) and urinary retention (UR) groups.

Clinical Parameters	Non-UR (n = 299)	UR (n = 37)	Univariate p-value	Multivariate p-value^+^
Age (yr)	68.3±6.6	69.2±5.6	0.443	
Body mass index (kg/m^2)^	24.0±2.9	24.5±2.3	0.391	
Symptom duration	27.2±43.1	22.2±37.0	0.502	
Comorbidity (n, %)				
Diabetes	49 (16.4)	5 (13.5)	0.816	
Hypertension	140 (46.8)	12 (32.4)	0.116	
Neurologic disease	32 (10.7)	3 (8.1)	0.781	
Cardiovascular disease	21 (7.0)	4 (10.8)	0.291	
Total prostate volume (ml)	56.0±24.2	53.2±18.8	0.424	
Transitional zone volume (ml)	30.2±19.9	26.4±15.3	0.385	
PSA (ng/dl)	3.5±4.2	3.2±2.6	0.711	
Urodynamic study				
First desire (ml)	195.9±77.6	200.1±62.7	0.615	
Normal desire (ml)	275.7±103.5	298.1±100.0	0.212	
Strong desire (ml)	371.1±113.8	379.2±100.4	0.378	
Maximal cystometric capacity (ml)	373.9±126.7	385.6±105.7	0.162	
PdetQmax (cmH_2_0)	61.1±27.5	58.5±24.5	0.886	
Bladder contractility index	83.3±44.6	76.2±28.6	0.345	
Bladder outlet obstruction index	44.5±29.4	41.6±26.5	0.688	
Operative parameters				
Operation time (min)	75.2±37.1	78.1±40.5	0.501	
Enucleation time (min)	56.1±24.3	58.0±31.7	0.277	
Enucleation efficiency (gm/mim)	0.4±0.3	0.4±0.2	0.652	
Morcellation time (min)	11.0±9.3	14.0±11.4	0.061	
Morcellation efficiency (gm/min)	2.1±1.3	1.6±0.8	<0.001	0.043(OR0.701,CI0.498–0.988)
Retrieved weight of prostate (gm)	20.9±17.2	20.3±15.4	0.572	
Intraoperative complication (n,%)*	92	19		
Bladder injury	22 (7.4)	5 (13.5)	0.336	
Capsular perforation	35 (11.7)	6 (16.2)	0.596	
Bleeding	68 (22.7)	13 (35.1)	0.153	
Postoperative complication (n,%)				
Reoperation due to bleeding (n,%)	1 (0.3)	4 (10.8)	0.001	0.005(OR0.039,CI0.004–0.383)
Transfusion (n,%)	3 (1.0)	3 (8.1)	0.020	0.036(OR0.144,CI0.027–0.877)
Catheterization duration (day)	2.0±1.7	2.9±3.2	0.106	
Hospital duration (day)	2.9±1.2	3.6±2.6	0.107	

PdetQmax, detrusor pressure at maximal flow; +, logistic multivariate analysis; all results are expressed as means ± SDs;

* , counts overlapped.

Morcellation efficiency was also found to be significantly different between the non-clot related UR and non-UR subjects (non-clot related UR 1.3±0.7 vs. non-UR 2.1±1.3 gm/min, p = 0.001). Obstructive IPSS score (clot-related UR 6.8±5.5 vs. non-UR 10.9±5.8, p = 0.005), reoperation due to bleeding rate (clot-related UR 3/24 vs. non-UR 1/299, p = 0.001), and transfusion (clot-related UR 3/24 vs. non-UR 3/299, p = 0.003) were significantly different in the clot-related UR group and the non-UR group (results not shown). In addition, obstructive IPSS score was significantly greater in non-clot related UR group than in the clot-related UR group (6.8±5.5 vs. 11.7±5.6, p = 0.023, data not shown in the table). To examine in more detail the risk factors of UR, we divided all 336 patients by bladder capacity into four groups (<200 ml, 201–400 ml, 401–500 ml, 500 ml≤), and examined the effects of medications taken before surgery by type (alpha blocker, anticholinergics, 5 alpha-reductase inhibitors) and the effect of surgical experience (0–20, 21–50, 51–100, and more patients). However, no significant differences in effects were found (p>0.05) (data not shown).

Comparisons of baseline IPSS scores, UFMs, and PVRs showed that significant improvements in all three variables in both non-UR and UR groups (p<0.05) (data not shown). However, non-UR group had significantly better clinical and objective outcomes than UR group after two weeks of operations. A total of 111 intraoperative complications were encountered, which included bladder injury, capsular perforation, and bleeding, but no significant intergroup differences were found (p>0.05). The mean total operation time was longer in the UR group than in the non-UR group, but this difference was not significant (UR 78.1±40.5 vs. non-UR 75.2±37.1 min, p = 0.501). No significant differences were observed with respect to catheter times (p = 0.106) or hospital stay (p = 0.107) (Table 3).

## Discussion

Over recent decades, many authors have demonstrated the efficacy, safety, and indications of HoLEP in LUTS/BPH as compared with other surgical procedures [15]. The advantages of HoLEP, such as, the absence of TUR syndrome, better hemostatic properties, lower perioperative morbidity, and shorter hospital stay are well established. Recently, HoLEP has been increasingly regarded as a new gold standard for treatment of LUTS/BPH [16], [17]. However, HoLEP still has its limitations, which can include a steep learning curve, diverse intraoperative, and early postoperative complications [18].

Failure to void after surgery is a difficult situation for both patients and clinicians. This study was designed to identify risk factors in UR patients after HoLEP by comparing these patients with non-UR patients, as knowledge of these factors might enable us to better understand the natural history, as well as risk factors, of UR. Previous studies have shown that complication rates are correlated with surgeon's experience [15], [19], [20]. However, in the present study, surgical experience was not found to influence UR.

Bladder over-distention has been previously reported to result in myogenic failure and detrusor instability in BPH patients [6], [12], [21]–[23]. In addition, some researchers claimed that the detrusor instability in diabetic patients [5]–[7] and the detrusor underactivity in BPH patients have also been associated with incidence of UR [7], [8], [24]. In this study, a history of diabetes was not associated with the UR. The bladder is distended to greater than maximal bladder capacity during the morcellation of enucleated prostatic nodule for an average morcellating time of 11.3 minutes and this is likely to adversely affect the bladder detrusor and result in postoperative voiding difficulties, especially de novo postoperative UR.

Other authors have reported rates of UR of 7–21% and clot-related UR of 0–5% after HoLEP. In the present study, these rates were 11.0% and 7.1%, respectively. After including all previously mentioned risk factors of UR [23], [25], [26], multivariate analysis showed that morcellation efficiency, reoperation, and transfusion were significantly independent risk factors for UR after HoLEP (Table 3). When we excluded clot-related UR population to identify risk factors of pure UR (non-clot related UR group), only morcellation efficiency was found to predict UR independently. Morcellation efficiency is associated with retrieved weight of prostatic nodules and morcellating time [14]. In the present study, retrieved weight of prostatic adenoma and morcellation time per se were not found to be significantly different between the non-UR and non-clot related UR groups (data not shown in tables). These findings suggest that some unknown factors might influence the development of UR. This may include the presence of hard nodules within the enucleated adenoma although we do not have data for this. In our opinion, hard nodules are assumed to be composed of dense fibrous stromal tissue. It tends to be very resistant to morcellation, which commonly make morcellation process unexpectedly prolonged and very difficult.

We considered the possible effect of bladder capacity of the patients on the postoperative UR, but no significant influence of the bladder capacity was found to be related with the prevalence of postoperative UR (data not shown). Regarding bleeding-related complications, multivariate analysis of the UR and non-UR groups showed that reoperation for bleeding control (OR 0.039, CI 0.004–0.383, p = 0.005) and transfusion (OR 0.144, CI 0.027–0.877, p = 0.036) were significantly associated with postoperative UR. Patients with a greater bleeding tendency resulted in a higher transfusion rate in the UR group than in the non-UR group.

In summary, we found that intraoperative careful bleeding control at the end of HoLEP, which prevent clot-related UR, is very important. Therefore, based on our results, we recommend meticulous intraoperative hemostatic coagulation during HoLEP. In addition, morcellation efficiency was found to be an independent risk factor, which might suggest that UR is related to intraoperative myogenic failure due to bladder over-distention. Therefore, it is also suggested that more efficient morcellation might reduce UR by minimizing potential injury to the detrusor muscle.

We sought to identify risk factors affecting the postoperative failure to void after HoLEP. To our knowledge, this is the first study to describe risk factors of UR for clot related and non-clot related UR (pure UR) in HoLEP patients. However, this study is limited by its retrospective design, and thus, a larger prospective study is needed in the future.

## Conclusions

The results reported in this study suggest that de novo postoperative UR after HoLEP is self-limiting. Patient age, history of diabetes, total operative time, and surgical experience were not found to be related to UR. Our findings also indicate that careful bleeding control during HoLEP procedure would help reduce the incidence of clot-related UR.

## References

[1] Gilling PJ, Cass CB, Malcolm AR, Fraundorfer MR (1995) Combination holmium and Nd:YAG laser ablation of the prostate: initial clinical experience. J Endourol 9: 151–153. 7633476 10.1089/end.1995.9.151

[2] Elzayat EA, Habib E, Elhilali MM (2005) Holmium laser enucleation of the prostate: a size-independent new “gold standard”. Urology 66: 108–113. 10.1016/j.urology.2005.06.006 16194716

[3] Hettiarachchi JA, Samadi AA, Konno S, Das AK (2002) Holmium laser enucleation for large (greater than 100 mL) prostate glands. Int J Urol 9: 233–236. 12060433 10.1046/j.1442-2042.2002.00457.x

[4] Suardi N, Gallina A, Salonia A, Briganti A, Deho F, et al (2009) Holmium laser enucleation of the prostate and holmium laser ablation of the prostate: indications and outcome. Curr Opin Urol 19: 38–43. 19057214 10.1097/MOU.0b013e32831a7008

[5] Montorsi F, Naspro R, Salonia A, Suardi N, Briganti A, et al (2004) Holmium laser enucleation versus transurethral resection of the prostate: results from a 2-center, prospective, randomized trial in patients with obstructive benign prostatic hyperplasia. J Urol 172: 1926–1929. 15540757 10.1097/01.ju.0000140501.68841.a1

[6] Eltabey MA, Sherif H, Hussein AA (2010) Holmium laser enucleation versus transurethral resection of the prostate. Can J Urol 17: 5447–5452. 21172109

[7] Tan A, Liao C, Mo Z, Cao Y (2007) Meta-analysis of holmium laser enucleation versus transurethral resection of the prostate for symptomatic prostatic obstruction. Br J Surg 94: 1201–1208. 17729384 10.1002/bjs.5916

[8] Peterson MD, Matlaga BR, Kim SC, Kuo RL, Soergel TM, et al (2005) Holmium laser enucleation of the prostate for men with urinary retention. J Urol 174: 998–1001. 16094022 10.1097/01.ju.0000170230.26743.e4

[9] Kuo RL, Paterson RF, Siqueira TM Jr, Watkins SL, Simmons GR, et al (2003) Holmium laser enucleation of the prostate: morbidity in a series of 206 patients. Urology 62: 59–63. 12837423 10.1016/s0090-4295(03)00124-9

[10] Elzayat EA, Habib EI, Elhilali MM (2005) Holmium laser enucleation of prostate for patients in urinary retention. Urology 66: 789–793. 16230139 10.1016/j.urology.2005.04.049

[11] Okamura K, Nojiri Y, Seki N, Arai Y, Matsuda T, et al (2011) Perioperative management of transurethral surgery for benign prostatic hyperplasia: A nationwide survey in Japan. Int J Urol 30: 304–310. 10.1111/j.1442-2042.2010.02712.x 21276084

[12] Bae J, Choo M, Park JH, Oh JK, Paick JS, et al (2011) Holmium laser enucleation of prostate for benign prostatic hyperplasia: seoul national university hospital experience. Int Neurourol J 15: 29–34. 21468284 10.5213/inj.2011.15.1.29 PMC3070223

[13] Kim M, Lee HE, Oh SJ (2013) Technical Aspects of Holmium Laser Enucleation of the Prostate for Benign Prostatic Hyperplasia. Korean J Urol 54: 570–579. 24044089 10.4111/kju.2013.54.9.570 PMC3773585

[14] Jeong CW, Oh JK, Cho MC, Bae JB, Oh SJ (2012) Enucleation ratio efficacy might be a better predictor to assess learning curve of holmium laser enucleation of the prostate. Int Braz J Urol 38: 362–371. 22765867 10.1590/s1677-55382012000300009

[15] Du C, Jin X, Bai F, Qiu Y (2008) Holmium laser enucleation of the prostate: the safety, efficacy, and learning experience in China. J Endourol 22: 1031–1036. 18377236 10.1089/end.2007.0262

[16] Elzayat EA, Elhilali MM (2006) Holmium laser enucleation of the prostate (HoLEP): the endourologic alternative to open prostatectomy. Eur Urol 49: 87–91. 16314033 10.1016/j.eururo.2005.08.015

[17] Elzayat EA, Elhilali MM (2006) Laser treatment of symptomatic benign prostatic hyperplasia. World J Urol 24: 410–417. 16518660 10.1007/s00345-006-0063-5

[18] Rieken M, EbingerMundorff N, Bonkat G, Wyler S, Bachmann A (2010) Complications of laser prostatectomy: a review of recent data. World J Urol 28: 53–62. 20052586 10.1007/s00345-009-0504-z

[19] Jeong CW, Oh JK, Cho MC, Bae JB, Oh SJ (2012) Enucleation ratio efficacy might be a better predictor to assess learning curve of holmium laser enucleation of the prostate. Int Braz J Urol 38: 362–372. 22765867 10.1590/s1677-55382012000300009

[20] Placer J, Gelabert-Mas A, Vallmanya F, Manresa JM, Menendez V, et al (2009) Holmium laser enucleation of prostate: outcome and complications of self-taught learning curve. Urology 73: 1042–1048. 19394500 10.1016/j.urology.2008.12.052

[21] Bae J, Oh SJ, Paick JS (2010) The learning curve for holmium laser enucleation of the prostate: a single-center experience. Korean J Urol 51: 688–693. 21031088 10.4111/kju.2010.51.10.688 PMC2963781

[22] Lane IF (2000) Diagnosis and management of urinary retention. The Vet Clin North Am Small Anim Pract 30: 25–57. 10680208 10.1016/s0195-5616(00)50002-3

[23] Shimizu N, Matsumoto S, Yoshioka N, Hanai T, Sugiyama T, et al (2006) Clinical study of acute urinary retention. Nihon Hinyokika Gakkai Zasshi 97: 839–843. 17154027 10.5980/jpnjurol1989.97.839

[24] Djavan B, Madersbacher S, Klingler C, Marberger M (1997) Urodynamic assessment of patients with acute urinary retention: is treatment failure after prostatectomy predictable? J Urol 158: 1829–1833. 9334611 10.1016/s0022-5347(01)64139-9

[25] Dubey D, Kumar A, Kapoor R, Srivastava A, Mandhani A (2001) Acute urinary retention: defining the need and timing for pressure-flow studies. BJU Int 88: 178–182. 11488724 10.1046/j.1464-410x.2001.02273.x

[26] Kuo HC, Chang SC, Hsu T (1993) Predictive factors for successful surgical outcome of benign prostatic hypertrophy. Eur Urol 24: 12–19. 10.1159/000474255 7689968

